# Effects of cognitive behavioural therapy for insomnia on the mental health of university students: study protocol for a randomized controlled trial

**DOI:** 10.1186/s13063-015-0756-4

**Published:** 2015-05-28

**Authors:** Daniel Freeman, Bryony Sheaves, Guy M. Goodwin, Ly-Mee Yu, Paul J. Harrison, Richard Emsley, Sophie Bostock, Russell G. Foster, Vanashree Wadekar, Christopher Hinds, Colin A. Espie

**Affiliations:** Sleep & Circadian Neuroscience Institute, Department of Psychiatry, University of Oxford, Warneford Hospital, Warneford Lane, Oxford, OX3 7JX UK; Nuffield Department of Primary Care Health Sciences, University of Oxford, Gibson Building 1st Floor, Radcliffe Observatory Quarter, Woodstock Road, Oxford, OX2 6GG UK; Centre for Biostatistics, Institute of Population Health, The University of Manchester, Manchester Academic Health Science Centre, 1.304 Jean McFarlane Building, Oxford Road, Manchester, M13 9PL UK; Sleepio Ltd, 60-62 Commercial Street, London, E1 6LT UK; Sleep & Circadian Neuroscience Institute, Nuffield Department of Clinical Neurosciences, University of Oxford, Level 5-6 West Wing, John Radcliffe Hospital, Oxford, OX3 9DU UK; Department of Psychiatry, University of Oxford, Warneford Hospital, Warneford Lane, Oxford, OX3 7JX UK

**Keywords:** Insomnia, Sleep, CBT, Delusions, Hallucinations, Schizophrenia

## Abstract

**Background:**

Insomnia, defined as repeated difficulties getting or staying asleep, is common in the general population. Such sleep difficulties are a problem in their own right, but increasingly it is being recognised that they may also be a contributory factor in the development of a wide range of mental health problems. Our focus is upon the relationship between insomnia and psychotic experiences, such as paranoia and hallucinations. Psychotic experiences commonly occur in mild forms in the general population and have been linked to disrupted sleep. These psychotic-like experiences raise the risk of development of a clinical disorder. Our aim is to reduce insomnia in a large general population group, and examine the effect on paranoia and hallucinations at the age when mental health problems typically emerge. The primary hypotheses are that cognitive behaviour therapy (CBT) for insomnia will reduce insomnia and also levels of paranoia and hallucinations. The theoretical links will be substantiated by a planned mediation analysis. Improvements in a number of other mental health outcomes are also predicted.

**Methods/Design:**

We will carry out a parallel group, randomised controlled trial of 2,614 students with insomnia in universities across the UK. In the Oxford Access for Students Improving Sleep (OASIS) trial, participants will be randomised to digital CBT for insomnia (in addition to treatment as usual) or treatment as usual. Online assessments will take place at zero, three, 10 (post-treatment), and 22 (follow-up) weeks. Primary outcomes are insomnia and psychotic-like experiences (paranoia or hallucinatory experiences) at 10 weeks. Secondary outcomes are levels of mania, depression, anxiety, nightmares, psychological wellbeing, and the development of mental health disorders. All main analyses will be carried out at the end of the last follow-up assessment and will be based on the intention-to-treat principle. The trial is funded by the Wellcome Trust.

**Discussion:**

This study will be the first large-scale causal test of the relationship between sleep disturbance and psychotic experiences. It will provide evidence concerning the clinical effects of treating insomnia in young adults.

**Trial registration:**

This trial was registered with Current Controlled Trials (identifier: ISRCTN61272251) on 29 January 2015.

## Background

Disrupted sleep is increasingly recognised as a contributory causal factor in the occurrence of many mental health problems [1–3]. This is based not only upon the knowledge that sleep promotes better daytime functioning, but that sleep processes and mental health disorders have overlapping mechanisms. Our particular interest is in the potential causal relationship between poor sleep and psychotic experiences, such as paranoia and hallucinations. A key approach to establishing a causal relationship, called an interventionist causal model [4], is to manipulate a putative causal factor and assess the effect on the outcome of interest. In the current trial we aim to improve sleep in people with insomnia in order to examine the effect on psychotic-like experiences. This approach therefore informs both theory and clinical practice.

### Insomnia

The most common form of sleep disruption is insomnia, which principally comprises sustained difficulties in initiating or maintaining sleep. Insomnia is a common psychological disorder: at any one time, approximately one third of the general population experience symptoms of insomnia [5, 6], whilst around 6 % of adults will currently meet diagnostic criteria for insomnia disorder [5, 7]. Just as for other mental health problems, there is a spectrum of severity in the general population. Although prevalence is high, natural remission appears to be low. In one patient study, 74 % of individuals continued to have insomnia one year later, and 46 % reported insomnia persisting for over three years [8]. Importantly, meta-analyses report moderate to large sustained improvements in insomnia following treatment with cognitive behavioural therapy (CBT) for insomnia [9–11]. Thus clinical guidelines recommend the use of CBT for the treatment of insomnia [12, 13].

### Psychotic experiences

A focus upon explaining individual psychotic experiences has gained increased interest because of evidence that the main diagnoses of psychosis, such as schizophrenia, schizoaffective disorder, and delusional disorder, do not capture single disorders. The empirical research indicates that within these diagnoses are multiple (relatively) independent experiences, such as paranoia, hallucinations, grandiosity, thought disorder, and anhedonia [14–16]. Furthermore, these problems are not confined to clinical disorders. There is a spectrum of severity in the general population of each of these individual psychotic experiences. For example, many people have a few paranoid thoughts, and a few people have many severe paranoid thoughts [17–19]. Whilst these psychotic like experiences are often transitory, their persistence over time constitutes a risk factor for developing later psychosis [20].

### Insomnia and psychotic experiences

In cross-sectional and longitudinal studies, paranoia in particular [21–24], but also hallucinations [25] have been linked to insomnia. Circadian rhythm disruption has been shown to be common in patients diagnosed with schizophrenia [26–28]. Importantly, a recent twin study of adolescents indicates that insomnia and psychotic experiences share overlap in genetic and environmental aetiology [25]. Applying the interventionist-causal model approach, we showed in a pilot case series that treating insomnia with CBT in patients with persecutory delusions (that is, severe paranoia) is associated with reductions in insomnia, paranoia, and anomalous perceptual experiences [29]. This is consistent with insomnia having a contributory causal role in psychotic experiences. Developing this work, we are completing a randomised controlled trial testing CBT for insomnia in 50 patients with delusions and hallucinations [30].

In the current trial we set out to test the effects of treating insomnia on psychotic-like experiences (and other mental health outcomes) in students attending university. A systematic literature search indicated that there had been no such similar tests. The benefit of choosing this participant group to test the hypotheses is twofold. First, it will enable recruitment of a large sample to test the hypotheses robustly. Circular emails will be sent at many UK universities, and students will be able to carry out the study online without face-to-face contact with the research team. Second, mental health problems such as psychosis typically have their first onset in late adolescence or /early adulthood [31], and therefore university students are at a vulnerable, and hence important, age to study. We will treat insomnia using the recommended approach of CBT. The CBT course will be delivered entirely online using computer or smartphone [32, 33]. It will be compared with treatment as usual, rather than an attention control or active control, because our central aim is to manipulate sleep to test the effects on mental health problems, that is, to conduct a mechanistic test. We therefore require one group to exhibit a change sleep patterns, and one group to show less change in sleep patterns. Furthermore, the CBT intervention to be used has already been shown to be of much greater efficacy than an attention control condition [32].

The primary hypotheses for the trial are:

1. The digital CBT intervention will reduce insomnia by the end of treatment (10 weeks).
2. The digital CBT intervention will reduce psychotic-like experiences (paranoia and hallucinations) by the end of treatment.
3. Changes in insomnia will mediate the changes in psychotic-like experiences.

The secondary hypotheses are:

1. The digital CBT intervention will reduce levels of depression, anxiety, nightmares, and mania by the end of treatment (10 weeks).
2. The digital CBT intervention will improve psychological wellbeing by the end of treatment.
3. The effects of CBT will be maintained at the scheduled follow-up assessment (22 weeks).
4. The digital CBT intervention will lead to the occurrence of fewer mental health disorders during the period of the trial, as assessed by screening tools at 22 weeks for ultra-high risk of psychosis, bipolar affective disorder, depression, and anxiety, and by treatment by mental health services.

## Methods/Design

### Research design

**Fig. 1:**
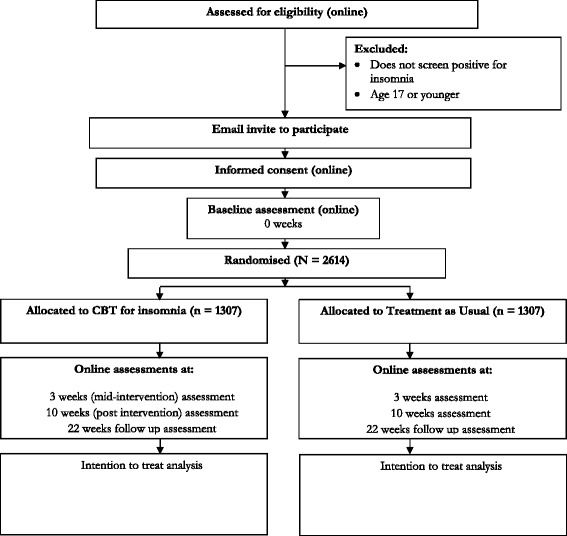
Summary of the trial design for the OASIS study

The study is a parallel group, superiority, randomised controlled trial of digital CBT in addition to treatment as usual versus only treatment as usual. The trial design is summarised in Fig. 1. The study will be carried out completely online. Participants will carry out screening, informed consent, assessments, allocation to condition, and intervention via a computer programme specifically designed for the trial. We will therefore run an internal pilot of 100 participants in order to check that the automated computer system is running correctly. The study has received ethical approval from the University of Oxford Medical Sciences Inter-Divisional Ethics Committee (reference number: MSD-IDREC-C2-2014-034). An information sheet is provided online, and informed consent is completed online before participation in the study can occur.

### Participants

We will recruit 2,614 students from UK universities. The inclusion criteria are: those with a positive screen for probable insomnia disorder, as indicated by a score of 16 or lower on the Sleep Condition Indicator (SCI) [34]; and being aged 18 or older. There are no exclusion criteria; we will not omit participants who report no signs of psychotic-like experiences because the dimensional measures of paranoia and hallucinations will be endorsed to some extent by a majority of participants, while we are also interested in other mental health outcomes that will be relevant to all participants (for example psychological wellbeing). Universities across the UK will be approached to take part. At each university, participants will be recruited via email circulars, which will provide a link to the web-based screening. Therefore many thousands of students at each university taking part will receive an invite to take part in screening for the study. Participants will not be recruited via health settings.

### Randomisation and allocation concealment

This study will use simple randomisation with an allocation ratio of 1:1, as recommended for large clinical trials [35]. It will be carried out by the automated online system, hence the research team will be unable to influence randomisation, and have no access to future allocations.

### Blinding

This will be a single blind trial. Assessments will be self-reported and carried out online, and hence the research team will be blind to outcomes during the trial. Participants will be informed of their randomisation outcome by an automatic email, and hence they will not be blind to treatment allocation. It is notable that the research team is unlikely to have any contact with research participants, and will therefore be unable to bias the allocation or assessments. If participants do contact the team and reveal the allocation, the absence of assessments carried out by an assessor prevents assessments becoming unblinded.

### Assessment points

Assessments will take place at the key assessment points of 0 (baseline), three (mid-therapy), 10 (end of therapy), and 22 weeks (follow-up). As an ethical consideration, all the control participants will be provided with CBT at 23 weeks, once the 22-week follow-up assessment has been conducted. We will therefore take this opportunity to include a further follow-up at 33 weeks for all trial participants (providing a potential replication of the within group treatment effect).

### Planned intervention

The CBT for insomnia intervention is delivered predominately via the internet. The delivery is structured into six sessions, lasting an average of 20 minutes each. The course takes a minimum of six weeks to complete, with sessions unlocked weekly. Participants can move at a slower pace, for up to a maximum of 12 weeks. All participants have to at least start the programme online. Certain tools (such as sleep diaries and relaxation audios) can also be accessed using the web browser of any smartphone. All of the six core sessions, sleep diaries, relaxation audios, and the scheduling tool can also be accessed using an iOS app, but this is only an option for participants who have an iPhone®. The treatment content is based on CBT for insomnia manuals [36–38] and includes a behavioural component (sleep restriction, stimulus control, and relaxation), a cognitive component (paradoxical intention, cognitive restructuring, mindfulness, positive imagery, and putting the day to rest) and an educational component (psycho-education and sleep hygiene).

The programme is highly interactive, and content is presented by an animated virtual therapist. Participants make a time for the session and are prompted via email and/or SMS if they do not ‘attend’. Participants complete daily sleep diary information throughout the intervention, which are used by the programme to provide tailored, personalised help. Participants receive an email and/or SMS reminder each morning to prompt them to fill in their sleep diary. In addition, participants complete a short questionnaire at the beginning of therapy to set treatment goals. Throughout the course of therapy, participants have access to a moderated online community and an online library of information about sleep. Participants can view their online case file, which includes four sections: a progress review, a reminder of strategies to try out between sessions, an agreed sleep schedule, and a list of further reading. The system provides online analytics, which can be used to monitor adherence by assessing how many sessions were completed and the number of weeks to complete the course. Data from the CBT programme show that 90 % of participants complete the course within 10 weeks; participants will have access to the intervention for up to 12 weeks. We note that treatment as usual will actually comprise of no intervention at all for the majority of participants, since they are not being recruited from clinical services.

### Outcome measures

Participants will receive an email prompt to complete the assessments online. The order of the assessments will be consistent across all participants and all time points. If participants do not complete the questionnaire within two days they will receive an email reminder. The full battery of questionnaires comprises 118 questions in total. The week three assessment is briefer, including only the key measures, which total 44 questions.

The primary measure for insomnia is the SCI [34]. This is an eight-item measure. Scores can range from 0 to 32, with higher scores indicating better sleep. A clinical cut off of less than 17 has been shown to correctly identify 89 % of those with probable insomnia disorder. The measure has good internal consistency (Cronbach’s alpha = 0.86) [34]. We will use a version of the SCI that includes one additional question regarding early morning waking.

The primary measure for paranoia will be the Green *et al.* Paranoid Thoughts Scale (GPTS), Part B [39]. The GPTS assesses persecutory ideation and the timeframe used will be the past fortnight. The scale comprises 16 items, each rated on a one (not at all) to five (totally) scale. The persecutory ideas sub-scale has excellent internal consistency (Cronbach’s alpha = 0.90), good test re-test reliability (correlation coefficient = 0.81), and good convergent and criterion validity [39].

The Specific Psychotic Experiences Questionnaire (SPEQ) - Hallucinations sub-scale [16] will be the primary outcome for hallucinatory experiences. The scale is adapted from the Cardiff Anomalous Perceptions Scale [40], and comprises nine items measuring hallucinatory experiences across a range of sensory modalities and has good internal consistency, test re-test reliability, and convergent validity [16]. Participants will be asked to consider the period of the past fortnight and the scale used for each item is from zero (not at all) to five (more than once per day).

Secondary outcome measures for sleep include the Insomnia Severity Index [41, 42] and the Disturbing Dreams and Nightmare Severity Index [43]. We will also include in the assessment one question concerning chronotype from the Morningness-Eveningness Questionnaire [44]. A secondary outcome measure for psychotic-like experiences will be the 16-item version of the Prodromal Questionnaire [45]. A score of six or more has 87 % specificity and 87 % sensitivity to correctly classify ultra-high risk for psychosis mental states in a help-seeking sample [45]. The measures to assess affective symptoms will be the Patient Health Questionnaire (PHQ) nine-item version [46], the Generalised Anxiety Disorder (GAD) seven-item version [47], the PHQ four-item version for a brief mid-therapy assessment [48], and the Altman Mania Scale [49].

To test the hypothesis concerning the potential development of mental health disorders by 22 weeks, specifically meeting criteria for ultra-high risk for psychosis, bipolar affective disorder, depression, and anxiety, we will use established cut-offs on the Prodromal Questionnaire [45], Altman Mania Scale [49], PHQ [46], and GAD-7 [47], respectively. Participants will be asked at each online assessment time point (apart from week three) whether they are in contact with mental health services, have received a mental health diagnosis, take medication for a mental health problem, or are currently receiving psychological therapy. We will additionally administer the Warwick Edinburgh Mental Wellbeing Scale [50], the Work and Social Adjustment Scale [51], and gather information on the demographics of the sample.

### Assessment of safety

The likelihood of serious adverse events occurring during this trial is low since CBT for insomnia has not been reported to cause them. The intervention offered in the trial has previously been tested in a randomised controlled trial testing change in insomnia and no adverse outcomes were reported [32]. We will record the occurrence of any serious adverse events in trial participants, defined as: all deaths, suicide attempts, serious violent incidents, admissions to secure units, and formal complaints about the online intervention. Owing to the online nature of the assessments and intervention, it is unlikely that the research team will become aware of all such events. Adverse events are likely to come to our attention only if we are contacted by a trial participant. For participants concerned about their mental health, a list of UK support services is provided on the study website. If a participant makes contact via email or telephone then the clinical psychologist coordinating the trial can advise on appropriate clinical services.

### Statistical analysis and sample size

Descriptive statistics within each randomised group will be presented for baseline values. These will include counts and percentages for binary and categorical variables, and means and standard deviations, or medians with lower and upper quartiles, for continuous variables, along with minimum and maximum values and counts of missing values. There will be no tests of statistical significance nor confidence intervals for differences between randomised groups on any baseline variable. Data distributions and all model assumptions will be checked for all analyses. If model assumptions are not met, data will either be transformed or analysed using a non-parametric test, as appropriate.

The sample size was calculated on the basis of change in psychotic experiences, since there will be a much larger standardised mean difference for sleep than psychotic-like experiences. Based on the standard deviations observed from previous studies for the SCI (SD = 2.5) and the GPTS (SD = 10.4) [32, 52], a total sample size of 2,614 (1,307 per group) will provide 90 % power to detect a small effect size with a standardised mean difference of 0.15 in psychotic-like experiences (primary outcome), whilst accounting for a high level of expected attrition (40 %).

After randomisation, participants will be analysed according to their allocated treatment group, irrespective of what treatment they actually receive. We will endeavour to obtain full follow-up data on every participant to allow full intention-to-treat analysis but we will inevitably experience the problem of missing data due to withdrawal, loss to follow up, or non-response questionnaire items. Data analysis will be performed using a mixed-effect model for repeated measures. The model will include treatment group, time, treatment-by-time interaction, and baseline covariates. An unstructured correlation matrix will be used to model the within-subject error correlation structure. An appropriate contrast will be specified to test for treatment efficacy between randomised groups for the primary outcome. We will also perform various sensitivity analyses using imputation methods to test whether the results are robust enough to allow for different assumptions about the missing data. The results from the trial will be prepared as comparative summary statistics (difference in response rate or means) with 95 % confidence intervals. All the tests will be done at a 5 % two-sided significance level. The study results will be reported in accordance with the Consolidated Standards of Reporting Trials (CONSORT) 2010 statements [53, 54] and Standard Protocol Item: Recommendations for Interventional Trials (SPIRIT) [55, 56] guidelines.

We will use modern causal inference methods for mediational analyses [57, 58]. This involves using parametric regression models to test for mediation of the digital CBT intervention on psychotic-like experiences through insomnia. Analyses will adjust for baseline measures of the mediator, outcomes, and putative measured confounders. We will include repeated measurement of mediators and outcomes to account for classical measurement error and baseline confounding, and instrumental variable methods (baseline covariate by randomization interactions as potential instruments) to investigate the sensitivity of the estimates to these problems and that of unmeasured confounding [57]. A full, detailed analysis plan, including plans for any interim analysis, subgroup analysis, and sensitivity analysis of the primary outcomes, will be prepared and finalised before the analysis.

## Discussion

There is growing interest in the role of sleep in the occurrence of mental health problems. The clinical implication is that improving sleep, using established interventions such as CBT, can lessen other mental health disorders. For example, a recent trial protocol describes a test of CBT to reduce the occurrence of depressive symptoms [59]. Our focus is upon sleep and psychotic experiences. The OASIS study has a strong mechanistic element, aiming to show the causal role of sleep in paranoia and hallucinations, as well as demonstrate the potential wide-ranging clinical benefits of the easily disseminated digital intervention. The study design benefits from being administered online, allowing us to recruit a large sample and limit researcher bias during the conduct of the study. It will, however, recruit a fairly homogenous sample of the population (university students), and this will limit the generalizability of the results. Nonetheless the study will still provide a strong test of the principal hypothesis that insomnia is a contributory causal factor in the occurrence of psychotic-like experiences. The CBT programme makes no reference to psychotic-like experiences and the techniques used are those designed to improve sleep; hence, there will be a clear manipulation of sleep, allowing causal inferences to made about the relationship to psychotic-like experiences.

## Trial status

Recruitment will begin in March 2015. It is anticipated that recruitment will be complete by March 2016, therefore trial results will become available in 2016.

## Abbreviations

CBT: Cognitive behaviour therapy
GAD: Generalised anxiety disorder (questionnaire)
GPTS: Green *et al.* paranoid thoughts scale (questionnaire)
PHQ: Patient health questionnaire
SCI: Sleep condition indicator (questionnaire)
SPEQ: Specific psychotic experiences questionnaire (questionnaire)
